# Role of SYT11 in human pan-cancer using comprehensive approaches

**DOI:** 10.1186/s40001-024-01931-3

**Published:** 2024-06-18

**Authors:** Kyunghee Noh, Hyunji Choi, Eun-Hye Jo, Wonbeak Yoo, Kyung Chan Park

**Affiliations:** 1 Bionanotechnology Research Center, Korea Research Institute of Bioscience and Biotechnology (KRIBB), Daejeon, 34141 Republic of Korea; 2 Department of Nanobiotechnology, University of Science and Technology (UST), Daejeon, 34141 Republic of Korea; 3 Personalized Genomic Medicine Research Center, Korea Research Institute of Bioscience and Biotechnology (KRIBB), Daejeon, 34141 Republic of Korea; 4 Department of Functional Genomics, University of Science and Technology (UST), Daejeon, 34113 Republic of Korea

**Keywords:** SYT11, Prognosis, Genetic alteration, Immune cell infiltration, Enrichment analysis

## Abstract

**Background:**

Synaptotagmin 11 (SYT11) plays a pivotal role in neuronal vesicular trafficking and exocytosis. However, no independent prognostic studies have focused on various cancers. In this study, we aimed to summarize the clinical significance and molecular landscape of SYT11 in various tumor types.

**Methods:**

Using several available public databases, we investigated abnormal SYT11 expression in different tumor types and its potential clinical association with prognosis, methylation profiling, immune infiltration, gene enrichment analysis, and protein–protein interaction analysis, and identified common pathways.

**Results:**

TCGA and Genotype-Tissue Expression (GTEx) showed that SYT11 was widely expressed across tumor and corresponding normal tissues. Survival analysis showed that SYT11 expression correlated with the prognosis of seven cancer types. Additionally, SYT11 mRNA expression was not affected by promoter methylation, but regulated by certain miRNAs and associated with cancer patient prognosis. In vitro experiments further verified a negative correlation between the expression of SYT11 and miR-19a-3p in human colorectal, lung, and renal cancer cell lines. Moreover, aberrant SYT11 expression was significantly associated with immune infiltration. Pathway enrichment analysis revealed that the biological and molecular processes of SYT11 were related to clathrin-mediated endocytosis, Rho GTPase signaling, and cell motility-related functions.

**Conclusions:**

Our results provide a clear understanding of the role of SYT11 in various cancer types and suggest that SYT11 may be of prognostic and clinical significance.

**Supplementary Information:**

The online version contains supplementary material available at 10.1186/s40001-024-01931-3.

## Introduction

Cancer is one of the leading causes of death worldwide [1] and considered an important factor affecting healthy human life and decreasing life expectancy. Therefore, a novel strategy to overcome cancer is one of the most urgent public health challenges. In the past few years, the advent of standard chemotherapy and supportive therapy has provided new ideas for cancer treatment [2–4]; however, the prognosis of patients with advanced cancer remains poor. Therefore, there is an urgent need to identify novel therapeutic targets with enhanced prognostic potential.

Synaptotagmin-11 (SYT11), a member of the synaptotagmin (SYT) family, is almost exclusively expressed in the brain tissue [5]. Recent studies have reported that SYT11 is a functional protein that binds calcium, phospholipids, or SNARE proteins throughout the neuronal cell body, axons, and dendrites and mediates vesicular trafficking [6, 7]. Genome-associated studies and experimental models have shown that SYT11 dysfunction is associated with Parkinson’s disease (PD) and susceptibility to schizophrenia in [8–12]. Interestingly, Bajaj et al. found that SYT11 plays a crucial role in tumorigenic properties such as invasiveness and metastasis in tumor microenvironment (TME) via Golgi-mediated exocytosis in lung cancer [13]. In addition, it is upregulated with SYT11 expression, associated with the stem-like molecular subtype of gastric cancer, and a prognostic biomarker for histologically classified diffuse-type gastric cancer [14]. However, owing to the importance of SYT11 in several cancers, new role of SYT11 in various cancers should be investigated.

Recently, there has been increasing focus on pan-cancer analysis in the context of tumor progression and prognosis. The Cancer Genome Atlas (TCGA) project is one of the most representative multi-omics data collection involving multiple cancers and it allows for the analysis of numerous genes in multiple cancers, including analysis of similarities and differences among genomic and cellular alterations for diagnostic, prognostic, and immunological parameters [15]. In this context, given the lack of an in-depth understanding of the role of SYT11, using pan-cancer analysis to investigate its roles in biological and pathological processes is an attractive approach, specifically to assess its value for cancer treatment.

In this study, we examined the expression profile and prognostic value of SYT11 in various cancer types. To further explore the aberrant patterns and possible clinical significance of SYT11 expression, correlation analysis was conducted between SYT11 expression and genetic alteration, methylation, miRNA interaction, and immune cell infiltration. Furthermore, interaction analysis of SYT11-correlated genes, protein–protein interaction (PPI), and functional enrichment analysis was also performed to explore their potential roles in biological and molecular processes. Based on a comprehensive analysis, we aim to provide a new understanding of the clinical value and clarification of SYT11 expression in various tumors.

## Materials and methods

### Data acquisition and processing

Gene expression profiling and interactive analyses based on TCGA and The Genotype-Tissue Expression (GTEx) information were obtained from GEPIA2 [16]. To further elucidate the overall survival (OS) and disease-free survival (RFS), we used the GEPIA2 database and further validated the data using the Kaplan–Meier Plotter (KM plot) [17]. The log-rank *p*-values and hazard ratios (HR) were automatically calculated. The cBioportal for Cancer Genomics database (https://www.cbioportal.org/) was used to analyze genetic alterations in *SYT11* in TCGA PanCancer Atlas Studies.

### Comprehensive analysis of SYT11 methylation

The SMART [18] was used to analyze the differential expression of methylated SYT11 in various cancers, and the UALCAN database [19] was used to analyze promoter methylation. All statistical analysis was automatically calculated online.

### Bioinformatic analysis of miRNA target prediction

To identify potential SYT11 targets, candidate miRNAs were predicted using miRWALK [20], TargetScan [21], and miRDB [22]. We screened 13 common miRNAs in miRWALK and miRDB based on the remaining miRNAs, excluding miRNAs without Pct values from TargetScan. The dbDEMC 3.0 database [23] was used to identify promising biomarkers for the expression levels of 13 candidate miRNAs in various cancers. Candidate miRNA expression correlation, prognosis, and enrichment analysis with SYT11 were conducted in pan-cancer using the Starbase database [24]. The criteria for miRNA interactomes were set such that the prediction program with miRanda–PicTar–TargetScan and suggested usage were selected.

### Exploring immune-related analysis in TME

The Tumor Immune Estimation Resource database (TIMER 2.0) was employed to investigate the correlation between SYT11 and tumor-infiltrating immune cells such as CD8 + T cells, macrophages, B cells, NK cells, MDSCs, and CAFs across diverse tumors from TCGA dataset [25]. The strength of the correlation heatmap with the purity-adjusted Spearman's ρ was statistically significant and automatically calculated online.

### Functional and pathway enrichment analysis of SYT11

The 24 genes with the strongest correlation with SYT11 were selected using Pathway Commons [26], database of publicly available information about biological pathways and biomolecular interactions, and this result was used to explore mRNA expression in various cancers. To further elucidate the functions and pathways, pathway-enriched analysis (including Reactome_2022 and BioPlanet_2019) and ontological analysis (including GO Biological Processes 2023 and GO Molecular Function 2023) for the 25 selected genes forming a cluster with SYT11 were performed using Enrichr [27] and the *p*-values were calculated online using Fisher’s exact test. Subsequently, the PPI network was evaluated using STRING to further understand the functions of interaction network for SYT11 protein. The strength and false discovery rates were automatically calculated online.

### Cell culture

HT-29, HCT-116, and Caki-1 cells were cultured in Dulbecco’s modified Eagle’s medium supplemented with 10% fetal bovine serum (FBS; GIBCO, Grand Island, NY, USA). CCD18co cells were cultured in Eagle's Minimum Essential Medium (EMEM, 30-2003, ATCC, Manassas, VI, USA) supplemented with 10% FBS and 1% penicillin–streptomycin. DLD-1, Colo 205, SNU482, H460, H441, and H1299 cells were cultured in RPMI 1640 medium supplemented with 10% FBS. All cells were grown in a humidified incubator with 5% CO_2_ at 37 ℃.

### Real-time quantitative reverse transcription polymerase chain reaction (qRT-PCR)

Total RNA was extracted using a mirVana™ miRNA Isolation Kit (Thermo Fisher Scientific, Waltham, MA, USA). cDNA was reverse-transcribed with the Verso cDNA Synthesis Kit for RT-PCR (Thermo Fisher Scientific) using oligo-dT primers for mRNA and stem-loop RT primers for miRNA, according to the manufacturer’s protocol. cDNA was amplified using previously reported primers (Supplementary Table 1) and SYBR Premix Ex Taq (Thermo Fisher Scientific). Relative mRNA and miRNA expression were analyzed by qPCR, according to the manufacturer’s instructions (Applied Biosystems, Foster City, CA, USA, and Agilent Technologies, Santa Clara, CA, USA), as we previously reported [Eur J Med Res. 2023 Nov 15;28(1):514]. β-actin was used as the endogenous control of mRNA expression, and U6 small nuclear RNA was used as the endogenous control for miRNA expression. Relative gene expression was quantified using the 2-ΔΔCT method. Pearson’s correlation analysis was performed to verify the relationship between the log_2_ expression values of the target miRNAs and mRNA.

## Results

### Differential SYT11 expression in various cancers

**Fig. 1:**
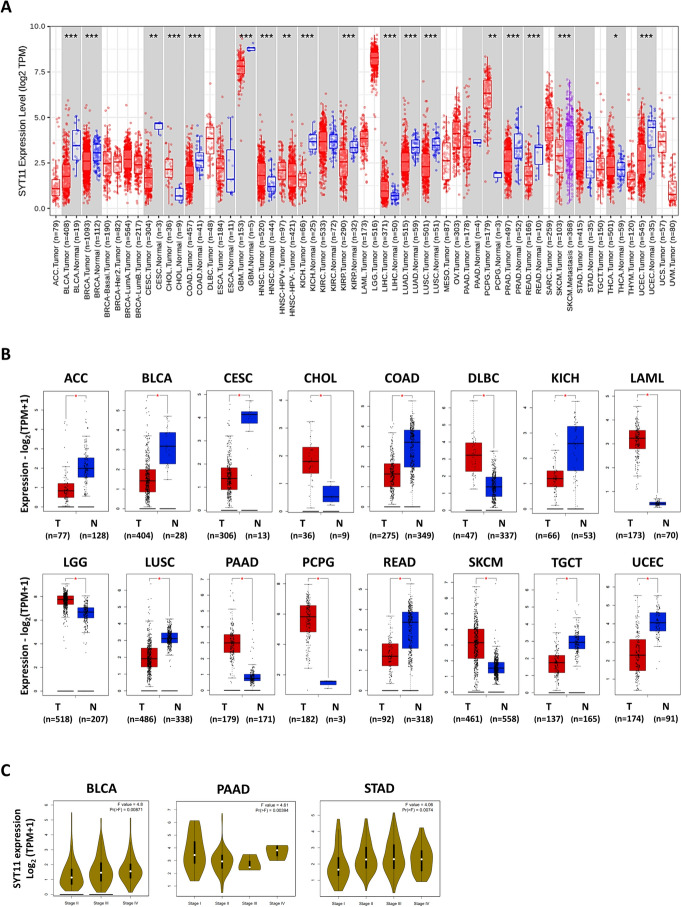
SYT11 expression level in various tumor tissues and stages. **A** The differences of SYT11 expression in various tumors or specific tumor subtype tissues and adjacent normal tissues analyzed by TIMER2 database from TCGA. *p < 0.05; **p < 0.01; ***p < 0.001. **B** Box plot representation of SYT11 expression level comparison in ACC, BLCA, CESC, CHOL, COAD, DLBC, KICH, LAML, LGG, LUSC, PAAD, PCPG, READ, SKCM, TGCT, and UCEC tumors relative to the corresponding GTEx database. *p < 0.05. **C** Pathological stage-dependent (stages I, II, III, IV, and V) SYT11 expression level. Expression in BLCA, PAAD, and STAD tumors were assessed and compared using TCGA data. Expression levels are shown as Log_2_ (TPM + 1)

According to the results obtained from the TIMER2 database, SYT11 was weakly expressed in most cancers, such as bladder urothelial carcinoma (BLCA), breast invasive carcinoma (BRCA), cervical squamous cell carcinoma and endocervical adenocarcinoma (CESC), colon adenocarcinoma (COAD), glioblastoma multiforme (GBM), renal hepatocellular carcinoma (KICH), kidney renal papillary cell carcinoma (KIRP), lung adenocarcinoma (LUAD), lung squamous cell carcinoma (LUSC), prostate adenocarcinoma (PRAD), rectum adenocarcinoma (READ), and uterine corpus endometrial carcinoma (UCEC) than in adjacent normal tissues, while being strongly expressed in cholangiocarcinoma (CHOL), head and neck squamous cell carcinoma (NHSC), liver hepatocellular carcinoma (LIHC), pheochromocytoma and paraganglioma (PCPG), and thyroid carcinoma (THCA) (Fig. 1A). Since TCGA database contains relatively insufficient information for normal tissues, we also included samples from the GTEx database for further analysis. SYT11 expression in the normal tissues of patients with adrenocortical cancer (ACC), BLCA, CESC, COAD, KICH, LUSC, READ, testicular germ cell tumors (TGCT), and UCEC is lower than the corresponding tumor tissues according to the GTEx database, while the pattern was opposite for the patients with CHOL, lymphoid neoplasm diffuse large B-cell lymphoma (DLBC), acute myeloid leukemia (LAML), brain lower grade glioma (LGG), pancreatic ductal adenocarcinoma (PAAD), PCPG, and skin cutaneous melanoma (SKCM) (Fig. 1B). However, SYT11 expression in the normal and tumor tissues of the patients with BRCA, ESCA (esophageal carcinoma), GBM, NHSC, KIRC, KRIP, LIHC, LUAD, ovarian serous cystadenocarcinoma (OV), PRAD, sarcoma (SARC), stomach adenocarcinoma (STAD), thyroid carcinoma (THYM), and UCS (uterine carcinosarcoma) were not significantly different (Supplementary Fig. 1). To investigate the SYT11 expression in detail, we generated a pathological stage plot using the GEPIA2 module. SYT11 RNA expression levels were significantly and positively associated with the late clinical stages of BLCA and STAD (Fig. 1C and Supplementary Fig. 2).

### Prognostic analysis of SYT11 in pan-cancer

**Fig. 2:**
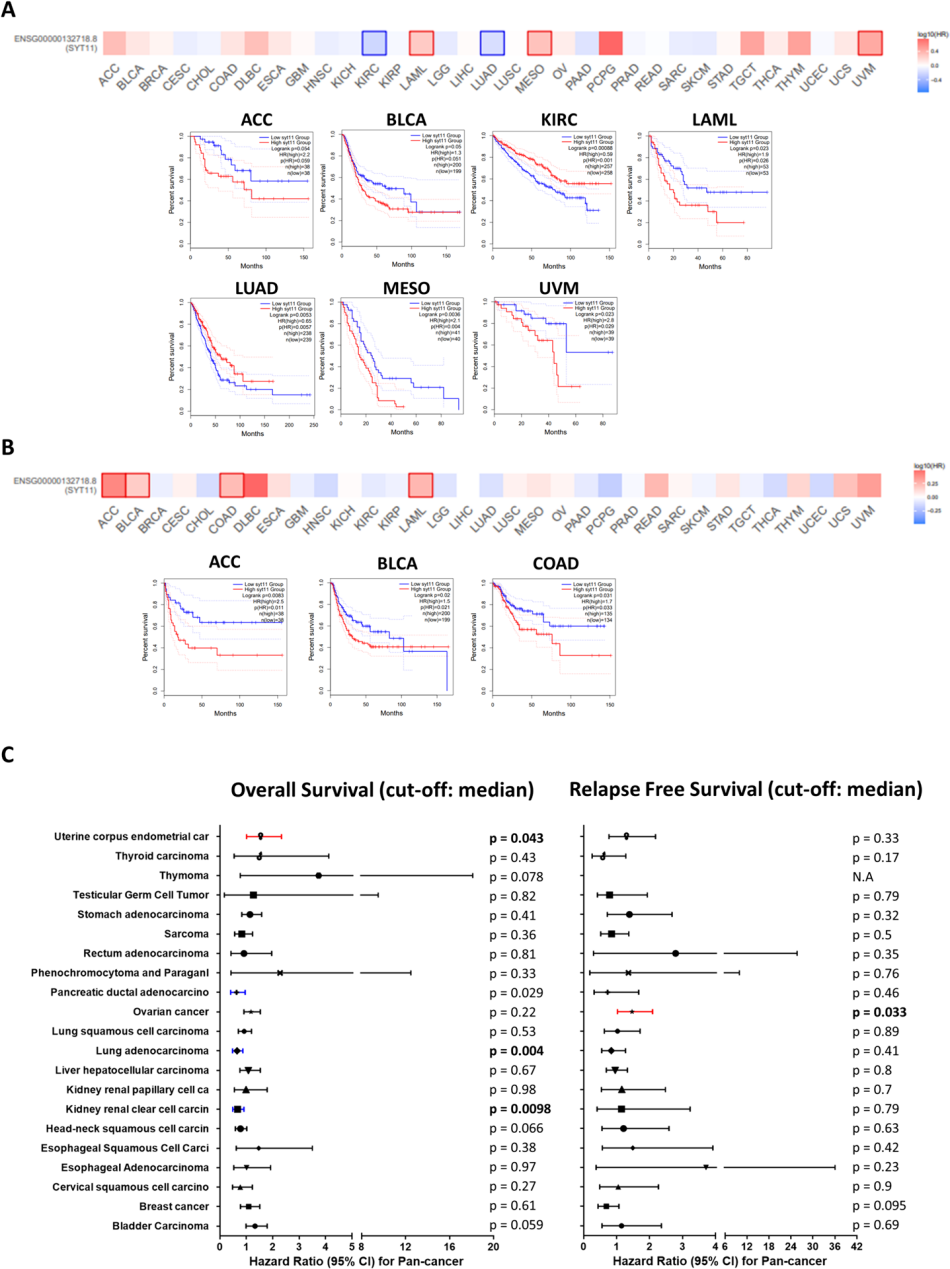
Correlation between SYT11 and prognosis in TCGA. **A** Overall survival analysis and (**B**) disease-free survival analysis in various cancer types from TCGA database. The survival map and graphs with positive results are displayed. **C** The forest diagrams of Kaplan–Meier plot analysis of overall survival and relapse-free survival according to SYT11 gene expression in TCGA data

**Fig. 3:**
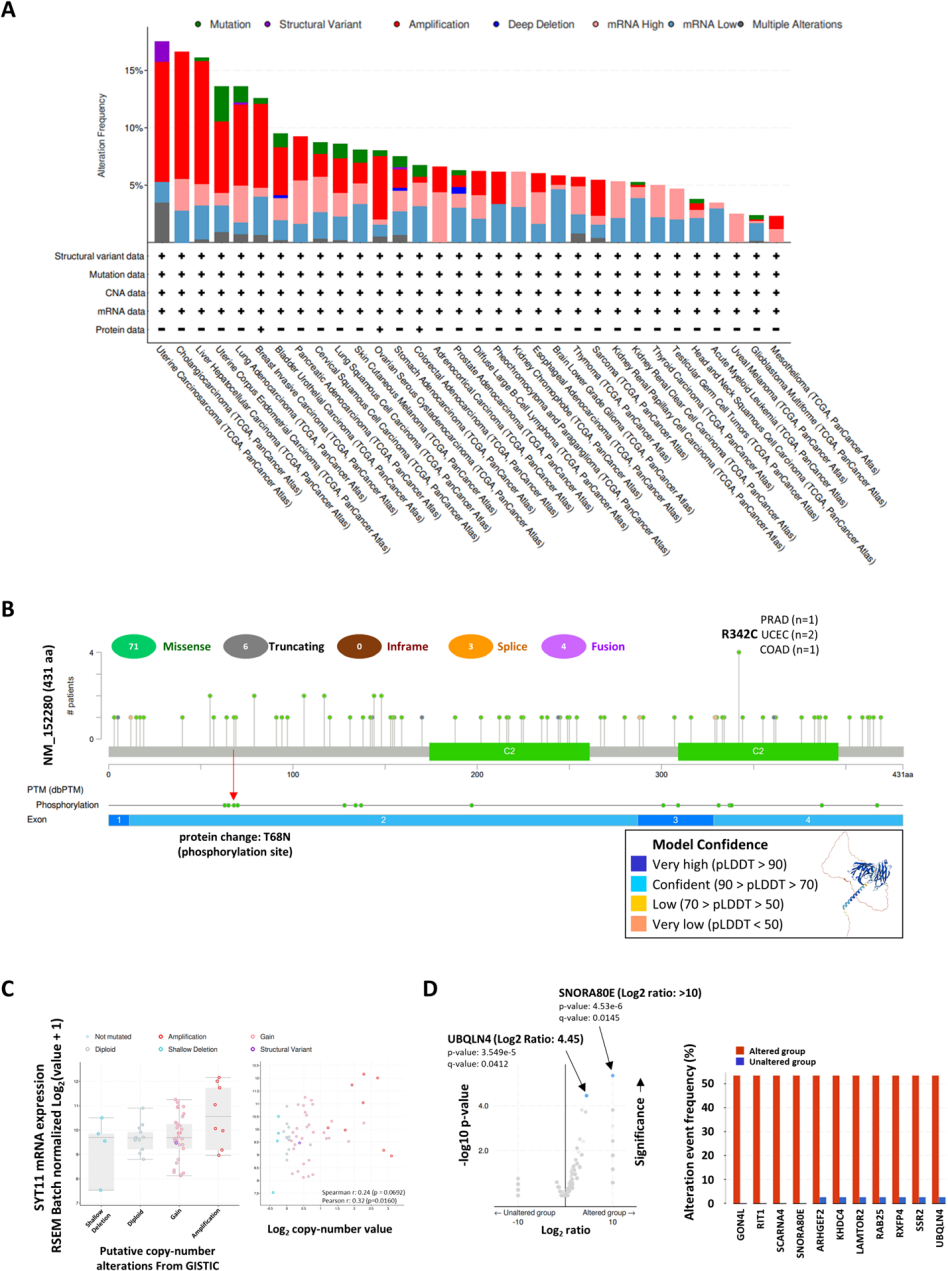
Genetic alterations of the SYT11 in pan-cancer. **A** Alteration frequency and (**B**) mutation and phosphorylation sites in SYT11. Analysis of clinical attributes in (**C**) putative copy number alterations, (**D**) molecular profiles on SYT11 genomic alteration in UCS. All data are from cBioportal database

To investigate the influence of SYT11 on the prognosis of various tumors, a heat map of the *SYT11* gene with significant prognostic value was generated using the GEPIA2 database, and the samples were divided according to the median SYT11 expression. Aberrant SYT11 expression mainly affected the improved prognosis of overall survival (OS) for patients with KIRC (*p* = 0.00088) and LUAD (*p* = 0.0053), whereas high SYT11 expression was associated with poor prognosis for patients with ACC (*p* = 0.054), BLCA (*p* = 0.05), LAML (*p* = 0.023), MESO (*p* = 0.0036), and UVM (*p* = 0.023) (Fig. 2A). Moreover, high SYT11 expression displayed unfavorable disease-free survival (RFS) for patients with ACC (*p* = 0.0083), BLCA (*p* = 0.02), and COAD (*p* = 0.031) tumors (Fig. 2B), but not others (Supplementary Figs. 3 and 4). Subsequently, we assessed the K–M plot to evaluate the relationship between SYT11 expression and prognosis of patients with different tumors. High SYT11 expression was associated with poor OS in patients with UCEC (*p* = 0.043), whereas patients with KIRC (*p* = 0.0098) and LUAD (*p* = 0.004) showed better OS (Fig. 3C). In addition, high SYT11 expression level was significantly associated with poor RFS in patients with OV (*p* = 0.033). To further substantiate the univariate analysis of prognostic SYT 11, we next performed a multivariate analysis regarding the OS. According to the multivariate cox regression model, stage was independent predictors of almost type of cancers, including ACC (HR 7.43, 95% CI 2.95–18.72), BLCA (HR 2.05, 95% CI 1.41–2.98), BRCA (HR 2.81, 95% CI 2.01–3.95), CESC (HR 2.12. 95% CI 2.01–3.95), COAD (HR 3.83, 95% CI 2.45–5.98), ESCA (HR 2.93, 95% CI 1.72–4.98), GBM (HR 1.02, 95% CI 1.01–1.04), HNSC (HR 1.94, 95% CI1.29–2.85), KICH (HR 10.69, 95% CI 2.07–55.07), KIRC (HR 3.82, 95% CI 2.79–5.24), KIRP (HR 6.23, 95% CI 3.23–12.04), LIHC (HR 2.49, 95% CI 1.71–3.61), LUAD (HR 2.5, 95% CI 1.83–3.43), LUSC (HR 1.58, 95% CI1.14–2.18), READ (HR 3.00, 95% CI 1.14–7.91), SKCM (HR 1.66, 95% CI 1.24–2.23), STAD (HR 2.16, 95% CI 1.52–3.07), THCA (HR 2.16, 95% CI 1.52–3.07), and UCEC (HR 4.12, 95% CI 2.67–6.37) (Supplementary Table 2).

### Genetic alteration of SYT11 in various cancers

Since genetic alterations are closely associated with tumorigenesis, the genetic variation of SYT11 in various cancers were determined using the cBioPortal TCGA cohort. As shown in Fig. 3A, alteration frequencies were high in the patients with UCS, CHOL, and LIHC, and the highest alteration frequency (> 10%) with ‘amplification’ in the patients with UCS. Accordingly, the most common alterations in SYT11 genes were missense (*n* = 71), truncating (*n* = 6), fusion (*n* = 4), and splice (*n* = 3) mutations, and the T68N mutation (Thr to Asn) was observed in the phosphorylation site. In addition, R342C was the main genetic alteration (one case in PRAD, two cases in UCEC, and one case in COAD) among the missense mutations (Fig. 3B). Based on the UCS showing the highest genetic alteration frequencies, we further analyzed the association between SYT11 and clinical attributes in UCS-TCGA. In the analysis of the putative copy number, SYT11 expression was the highest in the amplification group compared to that in the other groups, including shallow deletion, diploidy, and gain. Simultaneously, it was positively associated with copy number (Spearman *r* = 0.24, *p* = 0.0692; Pearson *r* = 0.32, *p* = 0.016; Fig. 3C). Regarding the association between SYT11 expression and copy number, we identified the molecular profiles of SYT11 genomic alterations. As shown in Fig. 3D, SNORA80E and UBQLN4 were significantly associated with SYT11 alteration as shown by volcano plots. Additionally, GON4L, RIT1, SCARNA4, SNORA80E, ARHGEF2, KHDC4, LAMTOR2, RAB25, RXFP4, SSR2, and UBQLN4 were significantly associated with SYT11 alterations.

### Difference of SYT11 methylation level in pan-cancer

**Fig. 4:**
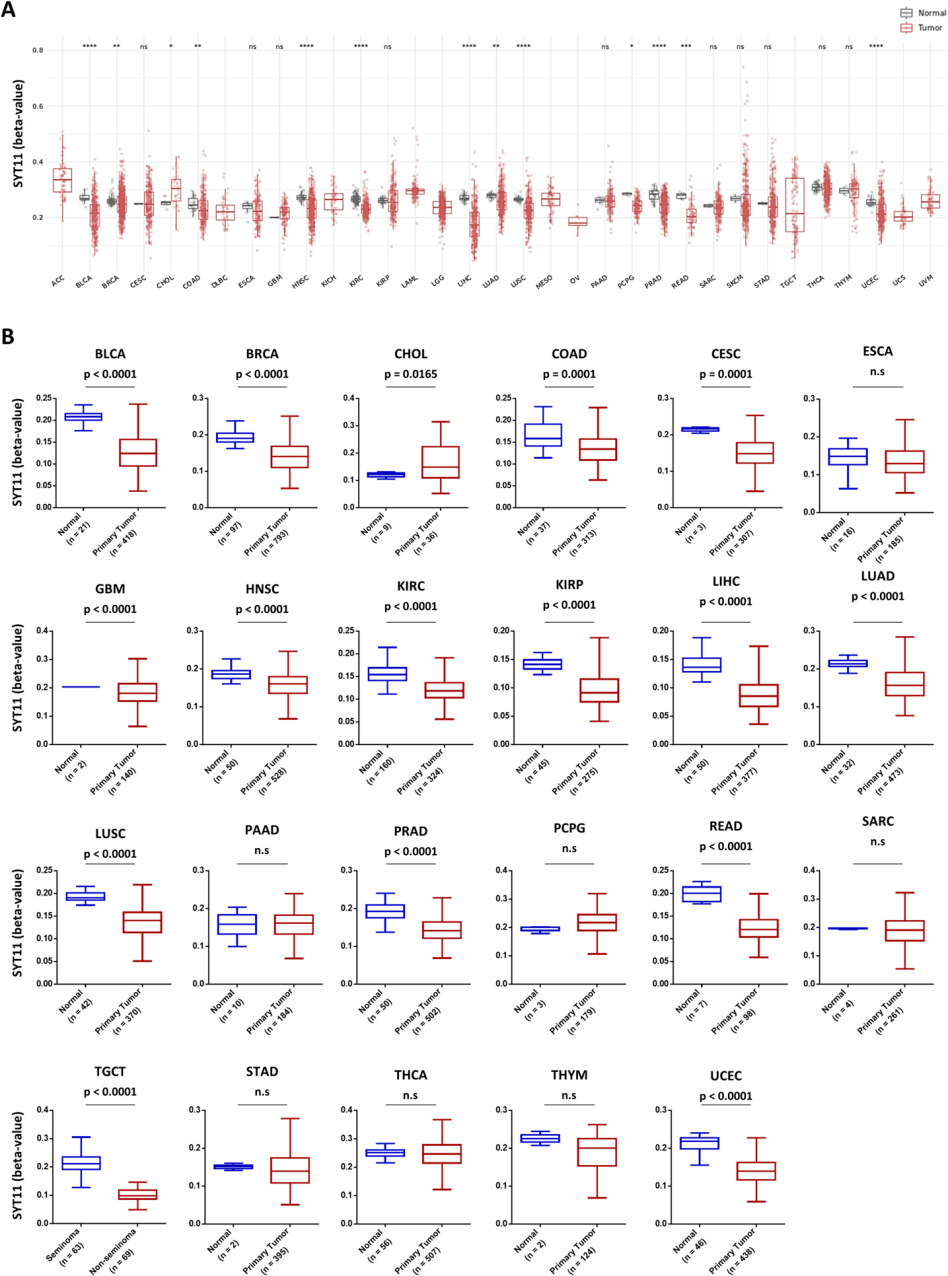
DNA methylation characteristics of SYT11. **A** SYT11 methylation levels in tumor and normal samples in patients with different cancer types. **B** The difference of promoter methylation between cancer and normal tissues. *p* > 0.05; *p ≤ 0.05; **p ≤ 0.01; ***p ≤ 0.001; ****p ≤ 0.0001. Ns, no significance

The Shiny Methylation Analysis Resource Tool (SMART) database was used to analyze the difference in SYT11 methylation levels between normal and primary tumor tissues. As shown in Fig. 4A, CpG-aggregated SYT11 methylation was significantly lower in tumor tissues than that in corresponding normal tissues for patients with BLCA (*p* ≤ 0.0001), BRCA (*p* ≤ 0.01), COAD (*p* ≤ 0.01), HNSC (*p* ≤ 0.0001), KIRC (*p* ≤ 0.0001), LIHC (*p* ≤ 0.001), LUAD (*p* ≤ 0.01), LUSC (*p* ≤ 0.0001), PCPG (*p* ≤ 0.05), PRAD (*p* ≤ 0.0001), READ (*p* ≤ 0.001), and UCEC (*p* ≤ 0.0001), while being the opposite for patients with CHOL (*p* ≤ 0.05). Since promoter methylation alters gene expression, we explored the promoter methylation level of SYT11 in tumor and normal tissues using the ULCAN database. The results showed that SYT11 promoter methylation was downregulated in patients with various tumors, including BLCA, BRCA, COAD, CESC, GBM, HNSC, KIRC, KIRP, LIHC, LUAD, LUSC, PRAD, READ, TGCT, and UCEC, but was lower in the primary tumor tissue then that in normal tissue only for the patients with CHOL. These results suggest that low SYT11 expression is less strongly associated with promoter methylation in most tumors.

### Prediction of SYT11 upstream miRNA and differential expression

**Fig. 5:**
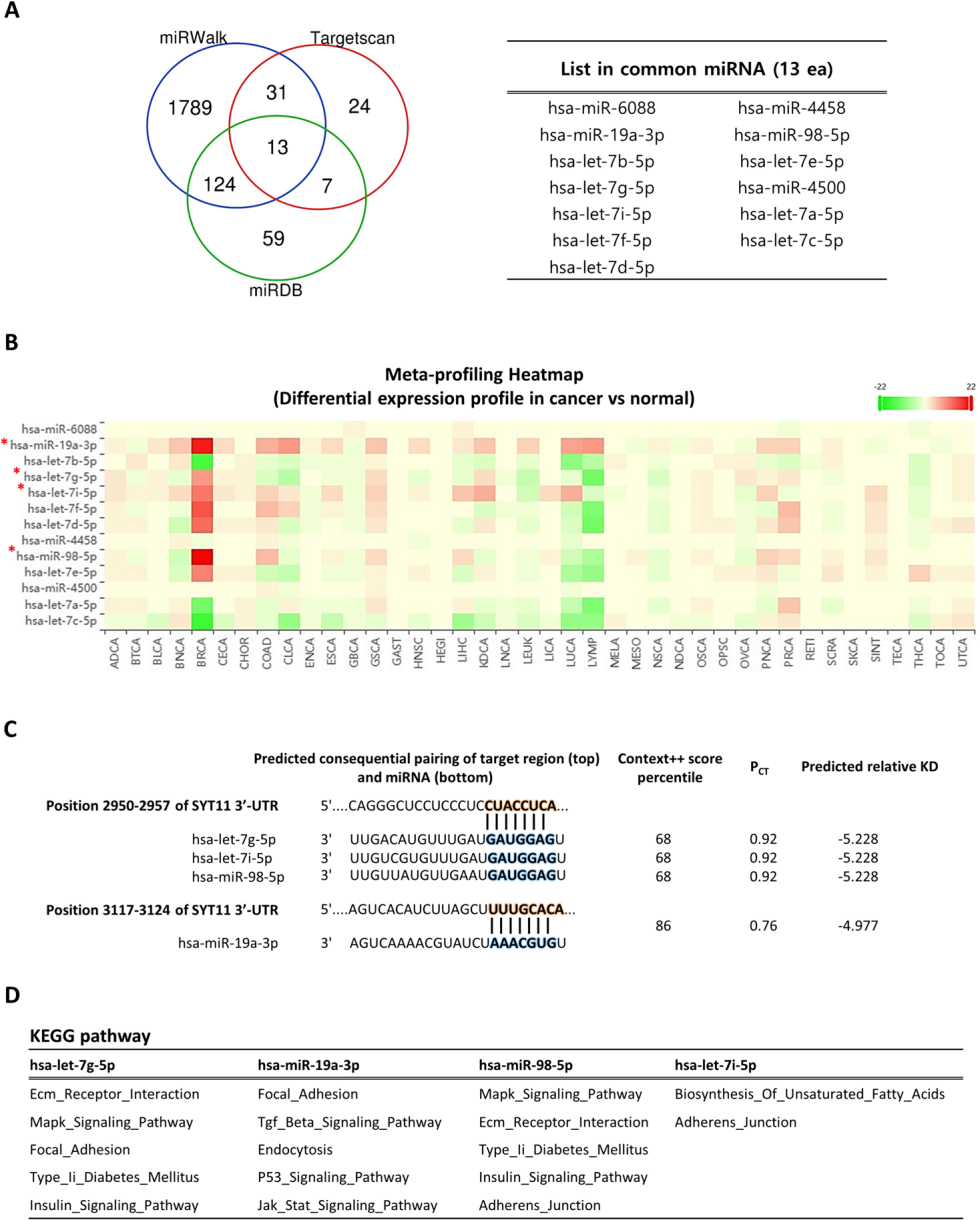
Screening of candidate miRNAs. **A** Venn diagram of miRDB, TargetScan, and miRWalk databases predicting miRNAs corresponding to SYT11 targets. **B** The differential expression meta-profiling heatmap of the 13 candidates, in cancer versus normal comparison, across various cancer types. **C** Predicted consequential pairing of miRNA target region. **D** The top five pathway of KEGG enrichment analysis in candidate miRNAs

**Fig. 6:**
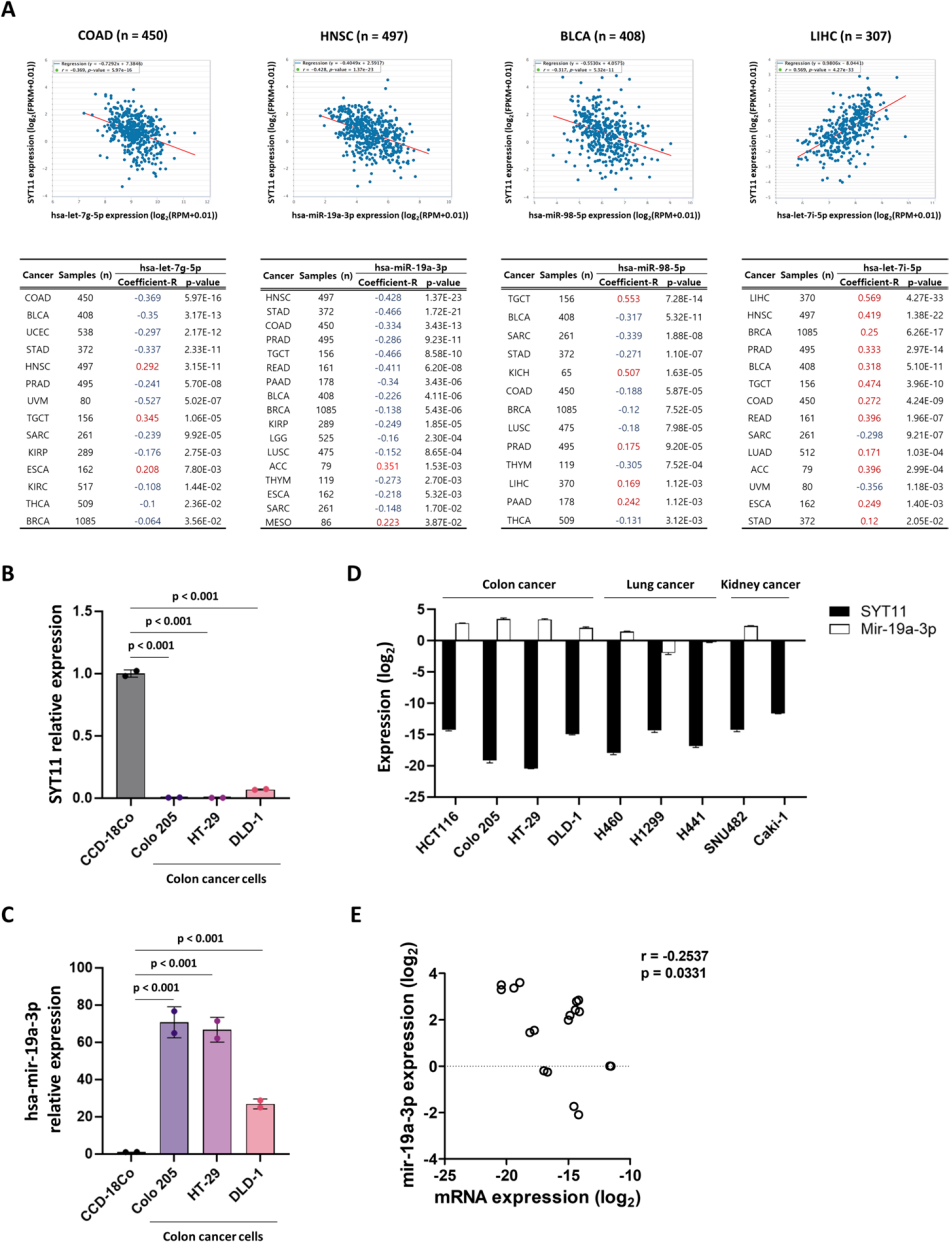
The association between SYT11 expression and candidate miRNAs. **A** SYT11 expression correlated with corresponding four target miRNAs (hsa-let-7g-5p, hsa-miR-19a-3p, hsa-miR-98-5p, and hsa-let-7i-5p) in various tumors. **B**–**D** qRT-PCR analysis of SYT11 mRNA and hsa-miR-19a-3p expression. **E** Correlation of SYT11 mRNA and miR-19a-3p expression; *p* values derived from the Pearson’s correlation

MicroRNAs (miRNAs) play crucial roles in post-transcriptional gene expression via base pairing within mRNAs. Since SYT11 expression is downregulated in various cancers, regulatory miRNAs are possibly highly expressed in cancer. To identify the target miRNAs of SYT11, we used miRNA prediction tools, including miRDB, TargetScan, and miRWalk, and then intersected 13 miRNAs by the Venn diagram (Fig. 5A). These 13 miRNAs were further analyzed to explore their differential expression profiles using a meta-profile heatmap of tissue samples from various cancer patients and healthy participants (Fig. 5B). Based on meta-profile heatmap, hsa-miR-19a-3p, hsa-let-7g-5p, hsa-let-7i-5p, and hsa-miR-98-5p showed significant differential expression in tissue samples of cancer patients and healthy participants, and presented binding sites with SYT11 3′-UTR (Fig. 5C). Simultaneously, biological network analysis showed that miRNA-mediated regulation was mostly enriched in intercellular signaling, environmental information processing, and cytoskeletal interactions, such as the MAPK signaling pathway, ECM receptor interaction, focal adhesion, and adherens junction (Fig. 5D and Supplementary Table 3). To further assess the relationship between expression and clinical significance, correlation analyses and Kaplan–Meier estimation were conducted between the four candidate miRNAs and SYT11 expression in pan-cancer samples. Among the miRNA/SYT11 pairs, hsa-let-7g-5p/SYT11, hsa-miR-19a-3p/SYT11, and hsa-miR-98-5p/SYT11 were negatively correlated with 11, 15, and 8 tumors, respectively. Conversely, the hsa-let-7i-5p/SYT11 pair was positively associated with most cancers (Fig. 6A). In terms of clinical survival prognosis, highly expressed hsa-let-7g-5p was linked to poor OS in the patients with THCA, COAD, SARC, and KIRC; hsa-miR-19a-3p in the patients with KIRC, THCA, SKCM, SARC, ACC, DLBC, BRCA, and LAML; hsa-miR-98-5p in the patients with TGCT, PRAD, ESCA, LGG, and HNSC; and hsa-let-7i-5p in the patients with KIRC, LGG, KIRP, and TGCT (Supplementary Fig. 5). Based on the above findings on SYT11 expression and important pan-cancer parameters, we selected colorectal cancer (CRC) for further analysis to validate the relationship between SYT11 mRNA and miR-19a-3p expression. To this end, we used the non-malignant human colon tissue-derived cell line CCD18co and the CRC cell lines Colo 205, HT-29, and DLD-1. As shown in Fig. 6B, C, SYT11 mRNA expression was downregulated in CRC cell lines compared to that in CCD18co cells; however, miR-19a-3p levels were significantly upregulated in CCD18co cells compared to that in CRC cell lines. In parallel with these findings, SYT11 mRNA levels were negatively correlated with miR-19a-3p levels in lung and kidney cancer cell lines (*r* =  − 0.2537; *p* = 0.0331) (Fig. 6D, E). These results indicate that the candidate miRNAs may play an important role in downregulating SYT11 expression and thereby affecting prognosis.

### Immune infiltration analysis of SYT11

**Fig. 7:**
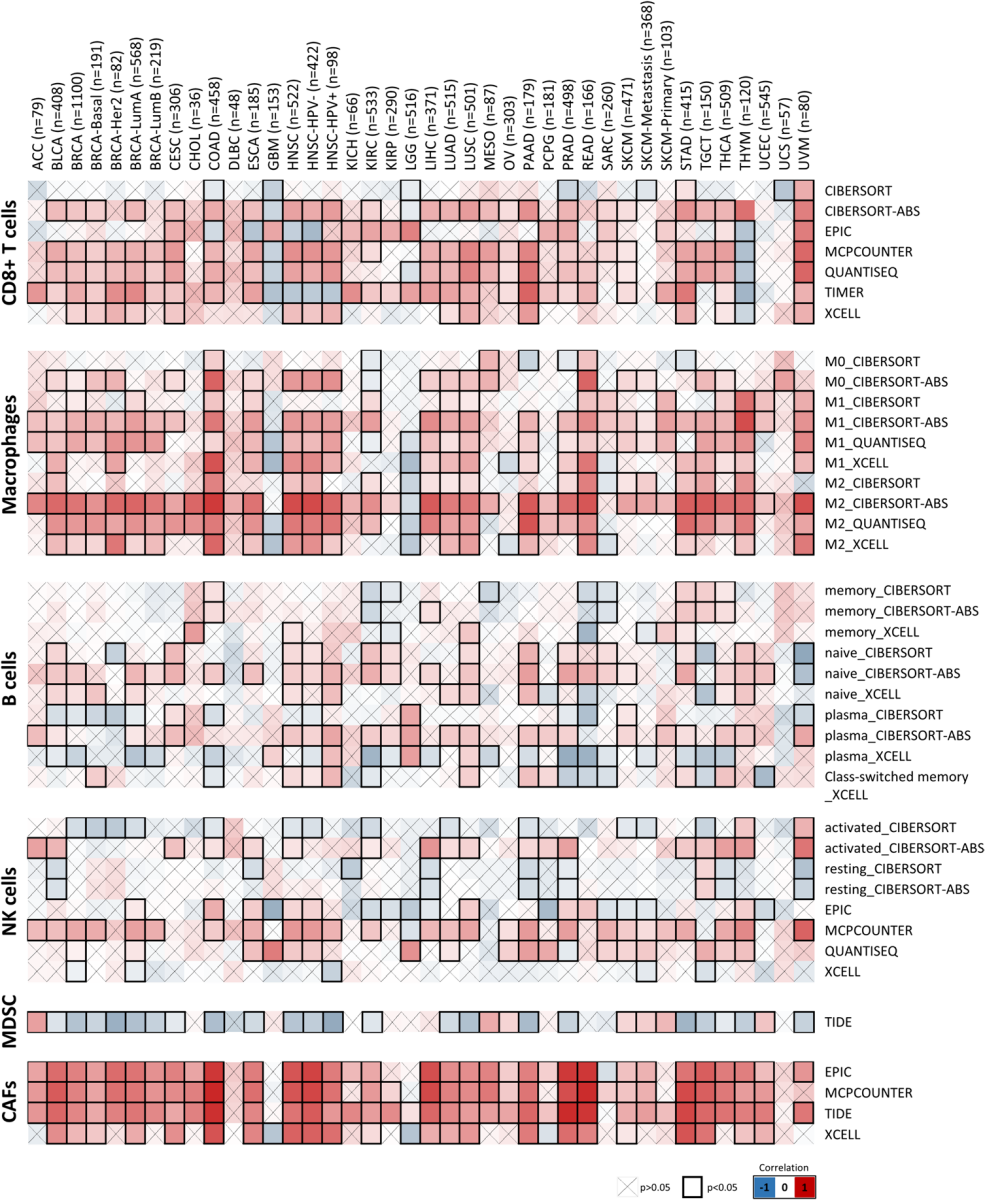
Correlation of SYT11 expression with immunological infiltration in various tumors

Since immune cell infiltration plays a crucial role in tumor progression, we investigated the relationship between SYT11 expression and immune cell infiltration in various tumors. As shown in Fig. 7, SYT11 expression was significantly positively associated with CD8 + T cell (in 14 types of cancer) and macrophage (in 13 types of cancer) infiltration. HNSC, LUSC, STAD, and THCA showed a positive tendency in B cells, but there was no clear trend in natural killer (NK) cells. Interestingly, SYT11 expression in myeloid-derived suppressor cells (MDSCs) showed a significant negative association with almost all cancer types, excluding ACC, MESO, OV, SKCM, and UCEC, while these negative correlations were associated with few CD8 + T cells and macrophages. In addition, SYT11 expression positively correlated with cancer-associated fibroblasts (CAFs) in most cancer types, except for DLBC, GBM, SARC, and UCS. These results suggest that SYT11 plays an important role in immune cell infiltration and may serve as a novel biomarker of various tumors.

### SYT11-related gene enrichment analysis data

**Fig. 8:**
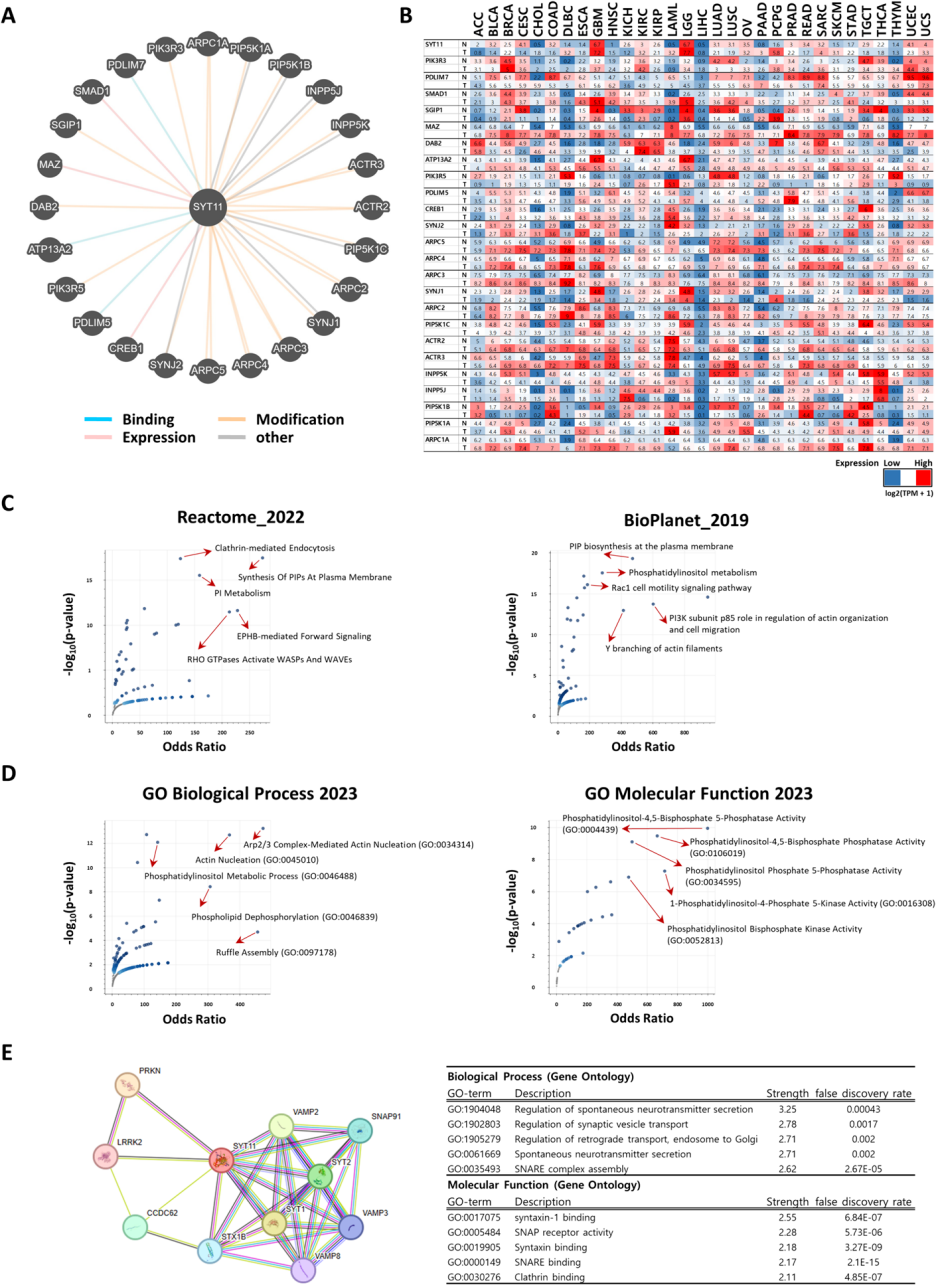
SYT11-related gene and protein enrichment analysis. **A** SYT11-interacting genes. **B** Expression analysis between SYT11-interacting gene in pan-cancers. **C** Pathway enrichment and (**D**) ontological analyses between SYT11-correlated gene in various tumors. **E** The protein–protein interaction (PPI) diagram to demonstrate the common differential expressed genes (DEGs). The top five lists contain identified biological and molecular process with a false discovery rate (FDR)

To further explore the potential mechanism of SYT11 in various tumors and clinical outcomes, we attempted to obtain a SYT11-interacted gene network (Fig. 8A). Twenty-four interacting genes and their expression profiles in various tumor and normal tissues are presented in Fig. 8B. Our results indicated that PDLIM7, SGIP1, DAB2, INPP5K, and PIP5K1B expression was higher in tumor tissues than that in the corresponding normal tissues, whereas the remaining interacting genes showed opposite tendencies. To assess the relationship between SYT11 and these genes, enriched pathway and ontological analyses were performed simultaneously. Pathway enrichment analysis revealed that SYT11 was significantly associated with clathrin-mediated endocytosis, phosphoinositide metabolism, and Rho GTPase activation in Reactom_2022 and phosphatidylinositol metabolism and cell motility signaling pathway in BioPlanet_2019 (Fig. 8C and Supplementary Table 4). In the ontological analysis, SYT11 was significantly linked with the cellular response to actin nucleation, phosphatidylinositol metabolism, and membrane ruffle formation in GO Biological Process 2023 and diverse phosphatidylinositol-based activities in GO Molecular Function 2023 (Fig. 8D and Supplementary Table 4). We also assessed the STRING database to obtain the SYT11-interacting proteins to support gene set enrichment analysis. As shown in Fig. 8E, SYT11 interact with 10 proteins, and these PPIs were further analyzed to explore their biological and molecular processes. The biological process results showed that SYT11-correlated proteins were involved in neurotransmitter secretion, synaptic vesicle transport regulation, and SNARE complex assembly. The molecular process results suggest that SYT11-correlated proteins are linked to syntaxin-1 binding, SNAP receptor activity, SNARE binding, and clathrin binding.

## Discussion

Recently, Bajaj et al. discovered a novel role for SYT11 in epithelial–mesenchymal transition (EMT)-mediated vesicular trafficking in the development of lung cancer invasion and metastasis [13]. In addition, SYT11 promoted the stem-like molecular subtype of diffuse gastric cancer [14]. However, whether SYT11 significantly impacts the pathogenesis of different tumors through common molecular mechanisms is not yet known. This study comprehensively explored the underlying molecular role of SYT11 in different tumor types and clinical prognoses using bioinformatics.

SYT11 mRNA expression analysis showed the possibility of predicting the diagnosis of certain tumors, such as COL, DLBC, LAML, LGG, PAAD, PCPG, and SKCM in TCGA, which showed high SYT11 mRNA expression and decreased expression in other tumors. Meanwhile, SYT11 expression was linked to diverse OS and DFS outcomes and poor prognosis in most highly expressed cancers. Although SYT11 expression does not perfectly align with survival prognosis, no reported studies have focused on tumors other than some lung and gastric cancers; therefore, the differential SYT11 expression is considered to be closely related to the survival prognosis of most tumors in this study.

Previous studies have demonstrated that multiple genetic and epigenetic events are highly involved in tumor initiation and progression [28–30]. Genetic alteration profiling of SYT11 revealed that amplification is the most common type of tumor, including UCS and missense mutations. In particular, GON4L, RIT1, SCARNA4, SNORA80E, ARHGEF2, KHDC4, LAMTOR2, RAB25, RXFP4, SSR2, and UBQLN4 were more frequently in the SYT11 altered group. Interestingly, these SYT11 co-occurring genes were enriched in pathways fundamental to cell function and metabolism, such as transcriptional regulation, cell survival, G protein-coupled signaling pathway, and ER function, which play an important role in tumor progression. In epigenetic analyses, such as promoter methylation profiling, SYT11 was hypomethylated in most tumor types than that in normal tissues, while the SYT11 expression was not consistent. Based on the inconsistencies between promoter methylation and mRNA expression, the association between miRNAs and gene expression was further investigated. Thus, hsa-miR-19a-3p, hsa-let-7g-5p, hsa-let-7i-5p, and hsa-miR-98-5p negatively regulates SYT11 and can interact with the SYT11 3’-UTR. Among the four miRNAs, hsa-let-7g-5p, hsa-miR-19a-3p, and hsa-miR-98-5p were negatively associated with SYT11 expression, whereas hsa-let-7i-5p was positively associated. All the predicted miRNAs were demonstrated to be involved in cell adhesion and proliferation, and related signaling pathways, such as the MAPK signaling pathway. hsa-miR-19a-3p and hsa-miR-98-5p are well studied miRNAs and have been reported to be involved in various cancer types and are potentially associated with prognosis [31–36]. Similarly, hsa-let-7g plays a tumorigenic role in lung cancer, osteosarcoma, and hepatocellular carcinoma [37–39], and high hsa-let-7i-5p expression is associated with kidney clear-cell carcinoma and CRC metastasis [40–42]. Importantly, we found that COAD, THCA, SARC, and KIRC were significantly correlated with miRNA–mRNA expression and OS (Fig. 6A, B). It was hypothesized that certain miRNAs other than the four predicted miRNAs, particularly hsa-let-7g-5p, may be related to epigenetic regulation and clinical prognosis in certain cancers. The reasons underlying the difference between epigenetic analysis and clinical outcomes in this study warrant further experimental investigation. Nevertheless, our findings provide useful information for further understanding the role of genetic and epigenetic SYT11 alterations.

Next, we visualized the immune infiltration landscape in various cancers, which are important TME components [43, 44]. Particularly, tumor-infiltrated B-cell is a prominent feature of the immune response to human cancer, suggesting the importance of strong prediction and prognosis for cancer therapeutics [45]. CAFs are activated fibroblasts with marked heterogeneity and plasticity in the TME and involved in tumor development, metastasis, and resistance to cancer immunotherapy. In addition, CAFs affect NK cell inactivation by inhibiting the cytolytic granule production signaling pathway, which causes cytotoxicity [46, 47]. The cytotoxic activity of NK cells plays a role in anti-tumor immunity through interactions with cancer cells, stromal cells, and extracellular substrates, specifically various surface molecules and metabolites [48]. Herein, we found that SYT11 expression is closely associated with immune components in various cancers and that SYT11 expression was weakly correlated with B-cell and NK cell immunity, but highly correlated with CAFs. Interestingly, we also found that SYT11 expression correlated more significantly with infiltration of M2 macrophages than that of M1 macrophages. M1 macrophages are tumor resistant macrophages as they can identify and kill cancer cells. In contrast to M1 macrophages, peri- and intra-tumoral M2 macrophages promote tumor progression through involvement in migration, invasion, angiogenesis, neovascularization, stromal activation, and extracellular matrix remodeling [49, 50]. Collectively, these findings suggest that abnormal SYT11 expression plays a role in the relationship between immune subsets and anti-tumor immunity.

In our analysis of SYT11-interacted genes, we determined the potential roles of clathrin-mediated endocytosis, Rho GTPase signaling, cell motility, and phosphatidylinositol metabolism. In the PPI network analysis, SYT-related proteins were highly enriched in the regulation of neurotransmitter secretion and transport in biological processes and the binding of syntaxin and clathrin in molecular function. Since Syt11 is an essential component of neuronal vesicle trafficking and synaptic plasticity [6] and a reliable EMT regulator in lung cancer invasion and metastasis [13], our study proves the above experimental results bioinformatically.

## Conclusion

This study is the first comprehensive pan-cancer analysis of SYT11 expression, including clinical prognosis, genetic alterations, epigenetic regulation, immune cell infiltration, gene enrichment analysis, and PPI network analysis, contributing to the clarification of the role of SYT11 from various perspectives in cancer. The critical role of SYT11 in cancer highlights its clinical value as a prognostic biomarker for various types of cancer. Importantly, to the best of our knowledge, we are the first to report the association of SYT11 expression with various components of the TME in multiple cancer types. Our findings provide new insights into the potential use of SYT11 as a prognostic biomarker and therapeutic target for cancer. Nevertheless, our study focused on bioinformatic analysis, and further basic and clinical validations are needed to confirm our findings. Further in-depth research is also required to address the issue of genetic and epigenetic discrepancies in the context of our results.

### Supplementary Information

Supplementary Material 1.

Supplementary Material 2.

Supplementary Material 3.

Supplementary Material 4.

Supplementary Material 5.

Supplementary Material 6.

## Data Availability

The original contributions of this study are included in the article and Supplementary Material. For further information, inquiries can be directed to the corresponding authors.
