# Supplementary material for: Role of SYT11 in human pan-cancer using comprehensive approaches
Source: Eur J Med Res. 2024 Jun 18;29:338. doi: 10.1186/s40001-024-01931-3 (PMC11186215; doi:10.1186/s40001-024-01931-3)

**Supplementary Figure 1** The differences of SYT11 expression in various tumors and adjacent normal tissues


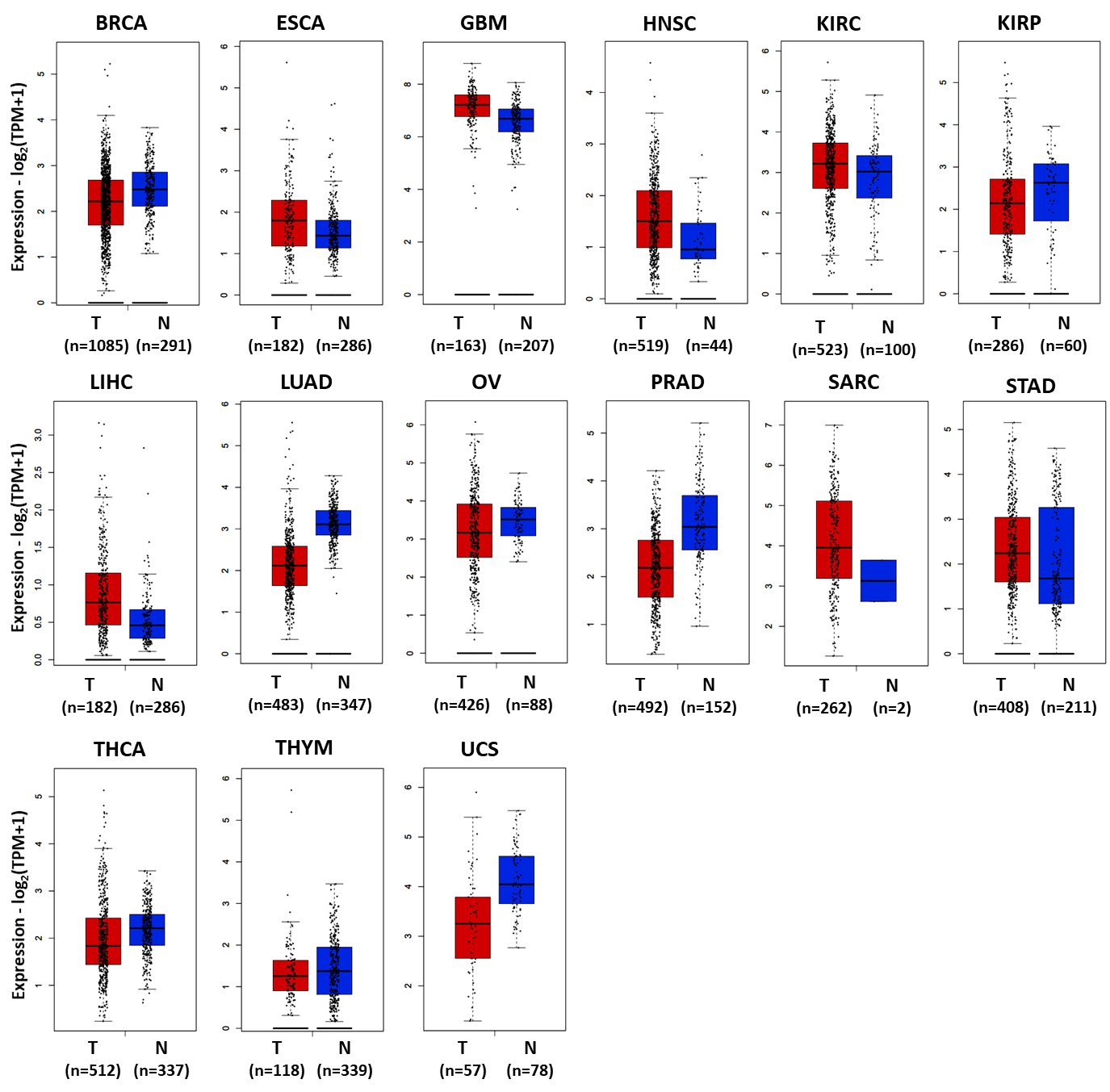


**Supplementary Figure 2.** Pathological stage-dependent (stage I, II, III, IV and V) SYT11 expression level in various tumors


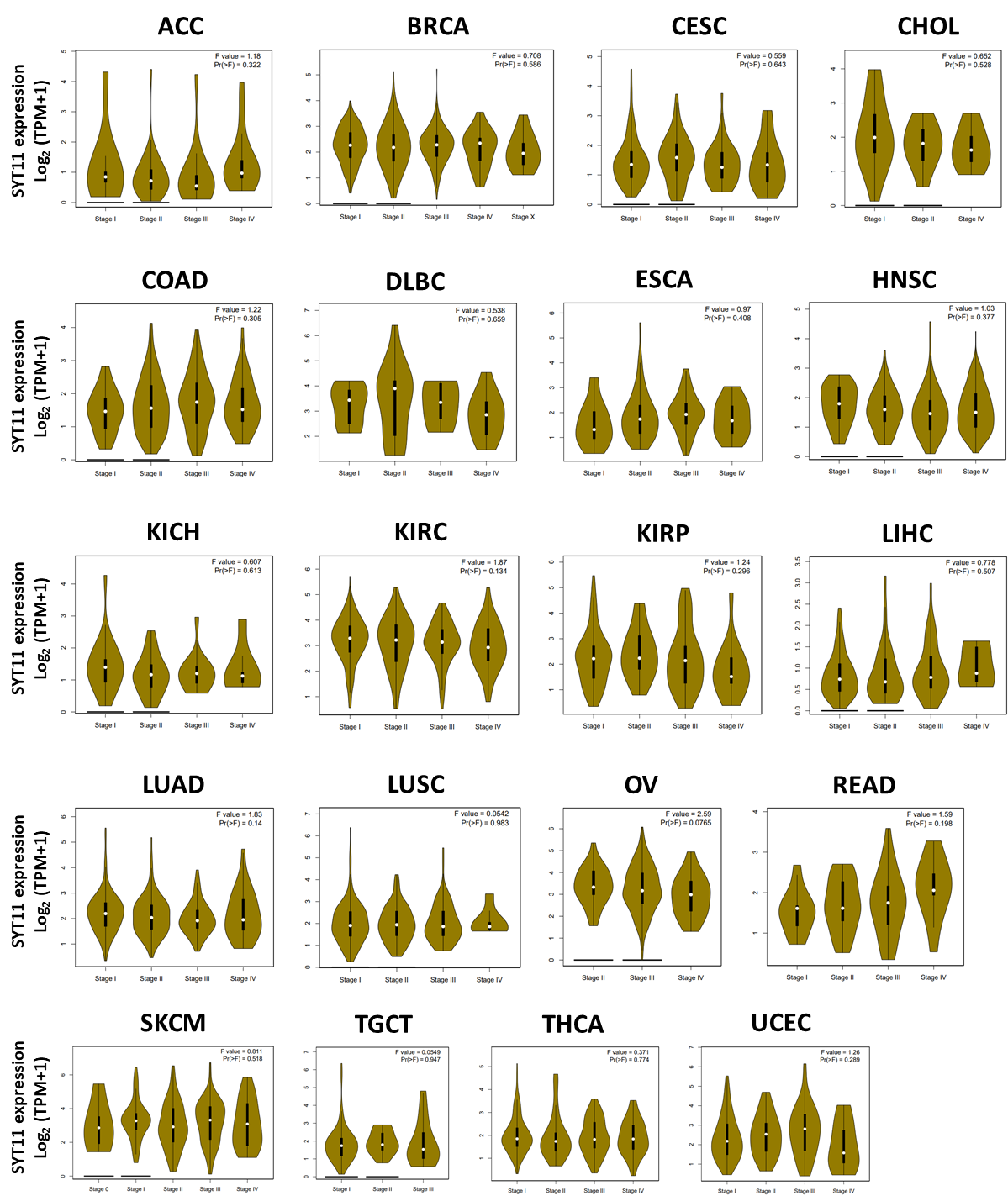


**Supplementary Figure 3** Overall survival analysis in various cancer types from TCGA database


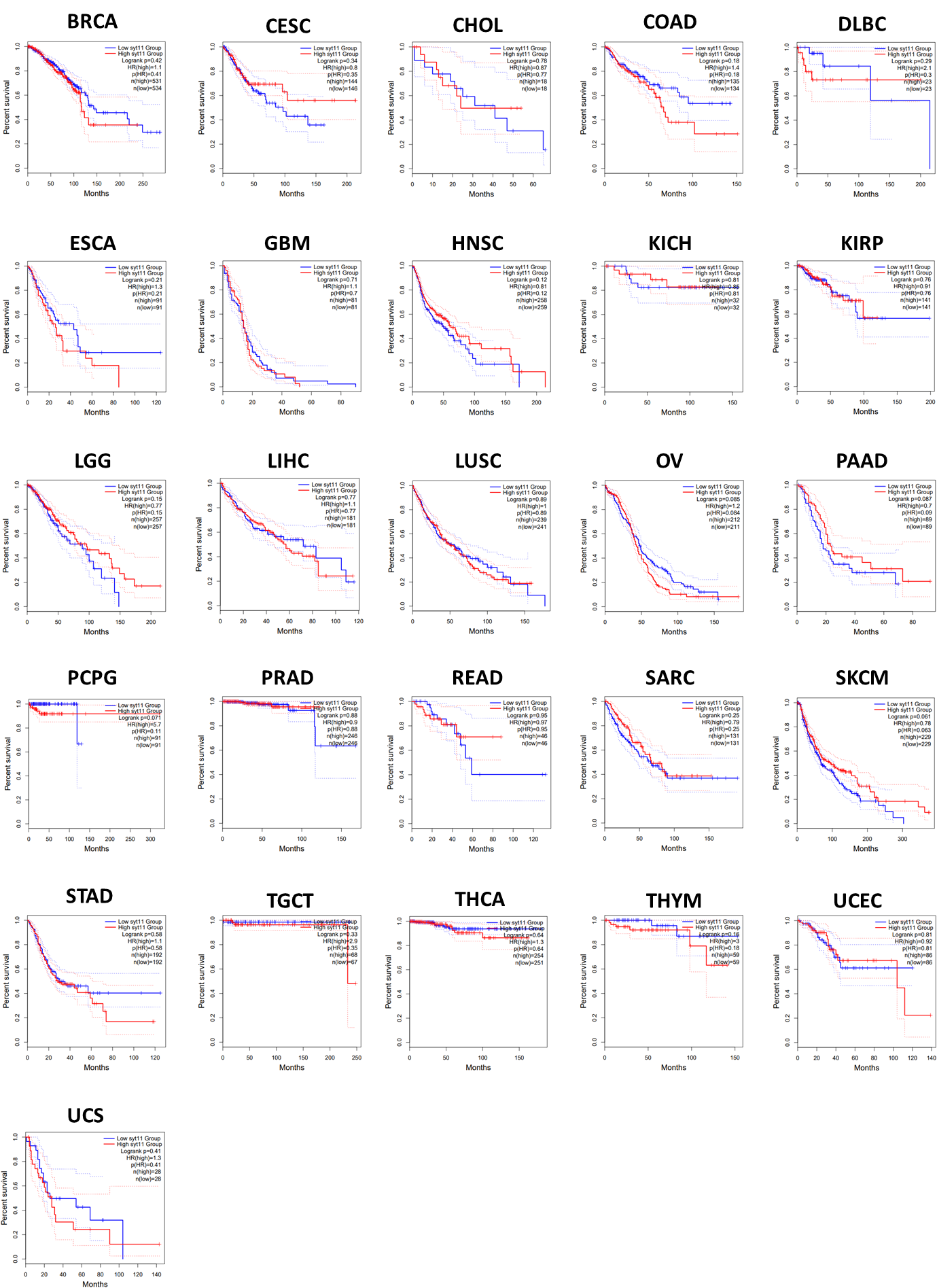


**Supplementary Figure 4** Disease-free survival analysis in various cancer types from TCGA database


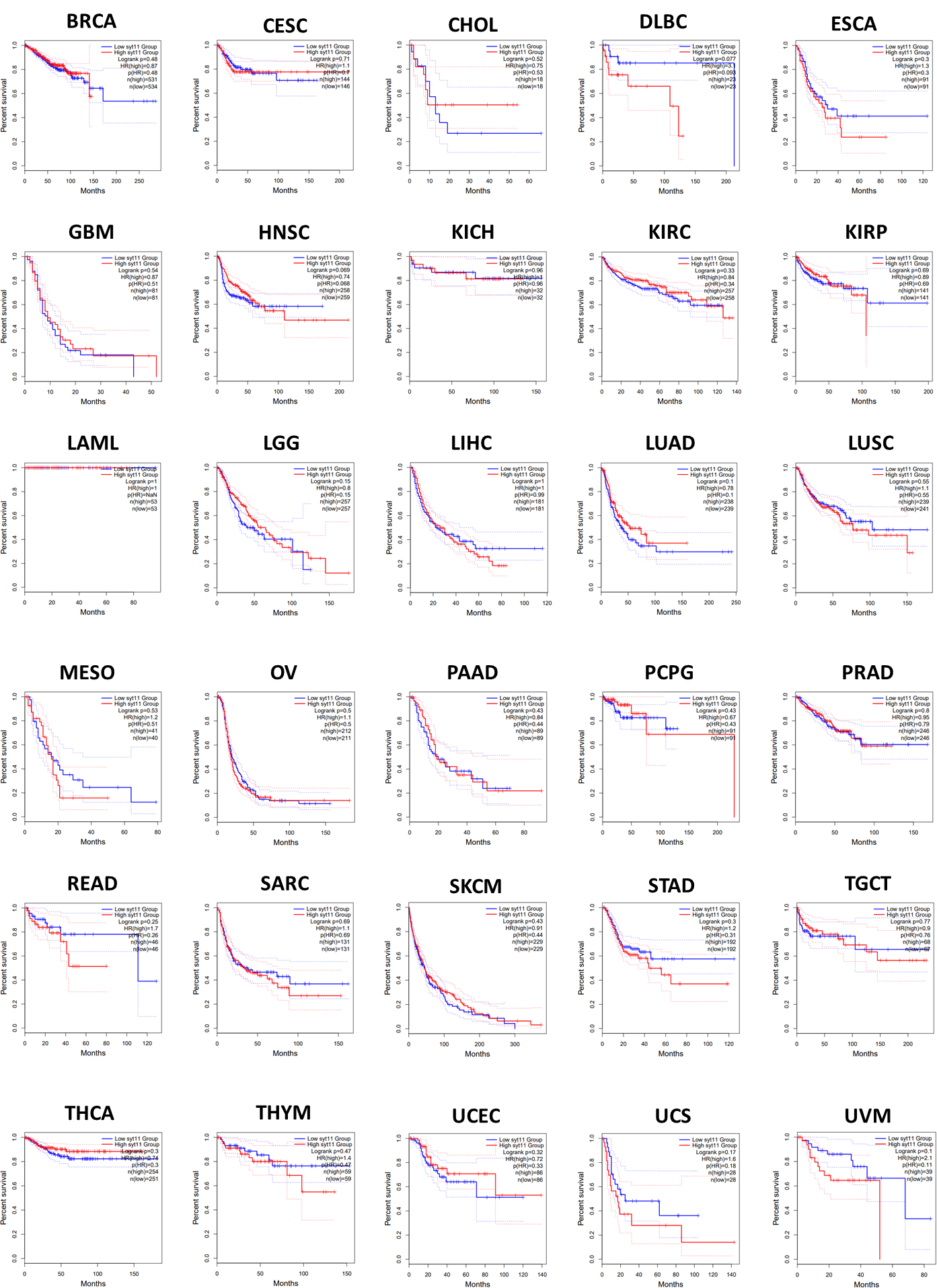


**Supplementary Figure 5** Correlation between candidate miRNAs and prognosis in various cancers.


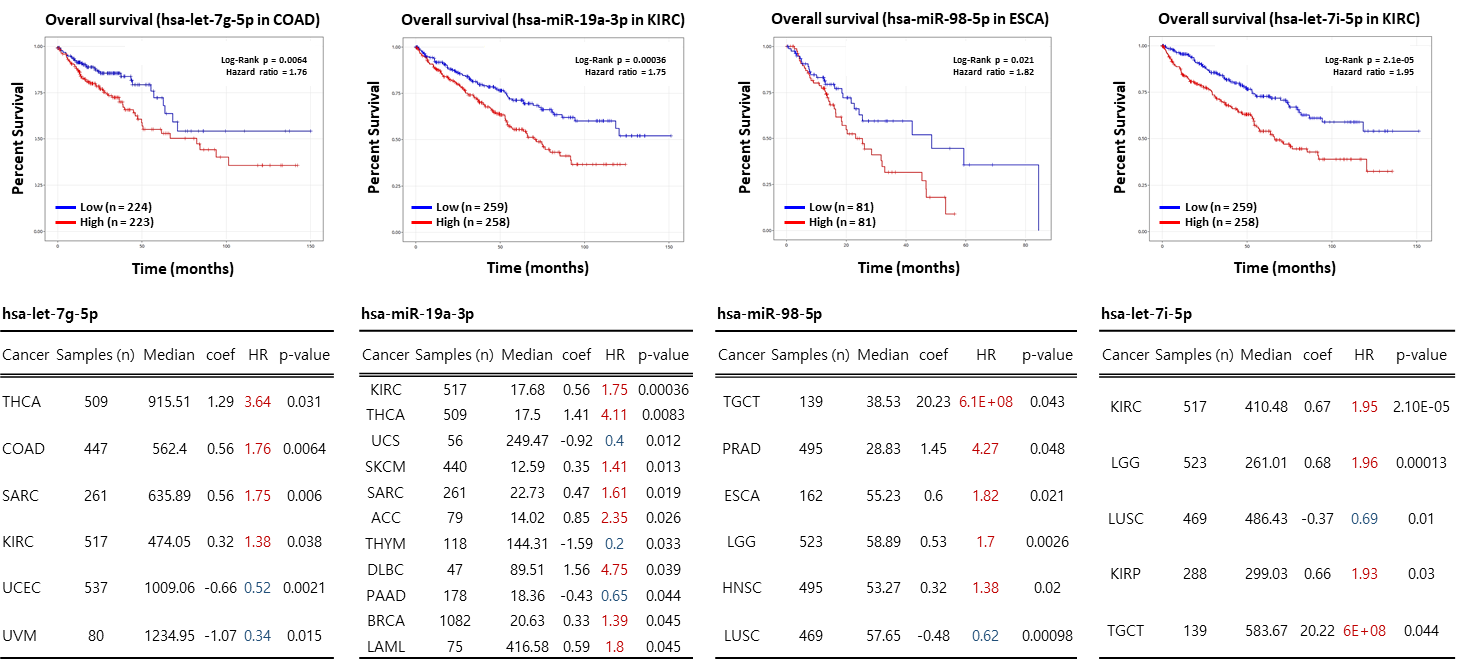

Supplement: Supplementary file 1 — Supplementary Material 1. [file 40001_2024_1931_MOESM1_ESM.docx]
