# Supplementary material for: Role of SYT11 in human pan-cancer using comprehensive approaches
Source: Eur J Med Res. 2024 Jun 18;29:338. doi: 10.1186/s40001-024-01931-3 (PMC11186215; doi:10.1186/s40001-024-01931-3)
Supplement: Supplementary file 2 — Supplementary Material 2. [file 40001_2024_1931_MOESM2_ESM.docx]

**Supplementary Table 5. Primer sequences for Stem-loop RT and qRT-PCR.**

| **Genes** | **Forward Primer (5’-3’)** | **Reverse Primer (5’-3’)** |
| --- | --- | --- |
| h*β-actin* | CTGTCCCTGTATGCCTCTG | TTGATGTCACGCACGATT |
| h*SYT11* | GTGGTCCTCAAAGCCAGACACT | GCAATGCGCTTTCTGCCGTAGT |
| *U6* | CTCGCTTCGGCAGCACA | AACGCTTCACGAATTTGCGT |
| *miR-19a-3p* | GGGTGTGCAAATCTATGCAA | TATGGTTGTTCACGACTCCTTCAC |
| **Genes** | **Primer (5’-3’)** | |
| *U6* RT | CTCAACTGGTGTCGTGGAGTCGGCAATTCAGTTGAGAAAAATATG | |
| *miR-19a-3p* RT | TGACCGTCTGTATGGTTGTTCACGACTCCTTCACCCTATCCAACCATACAGACGGTCATCAGTTTTG | |
